# Factors affecting the early post-operative prognosis in morbidly obese surgical patients after laparoscopic sleeve gastrectomy – a retrospective cohort study

**DOI:** 10.1186/s40981-017-0113-6

**Published:** 2017-08-31

**Authors:** Takashi Kobayashi, Yoko Watanabe, Jun Aizawa, Kenji S. Suzuki

**Affiliations:** Department of Anesthesiology, School of Medicine, Iwate Medical University, 19-1 Uchimaru, Morioka-shi, Iwate, 020-8505 Japan

**Keywords:** Morbid obesity, Sleeve gastrectomy, Anesthesia management

## Abstract

**Background:**

The number of morbidly obese patients who have undergone bariatric surgery has been gradually increasing in Japan. These obese patients are often complicated with metabolic, cardiac, respiratory, and other diseases. The aim of this study was to analyze the perioperative clinical course in a retrospective cohort with respect to the utility of anesthesia management in order to prevent longer hospital stays after surgery.

**Findings:**

Sixty-seven morbidly obese patients who had undergone sleeve gastrectomy were divided into two groups, based upon the duration of postoperative hospital stay; group S was comprised of the patients who were discharged within 5 days after surgery (*n* = 57) and group L was comprised of those who were discharged after 6 days or more (*n* = 10). The mean duration of the hospital stay was 4.8 ± 0.4 days and 7.8 ± 1.4 days in groups S and L, respectively. Multivariate logistic regression analysis showed that prolonged anesthesia was a predictor of a longer postoperative hospital stay (*p* < 0.05). While the difference in BMI was not significantly different, the percentage of patients with BMI ≥ 50 was 12 and 30% in groups S and L, respectively.

**Conclusions:**

Longer duration of anesthesia affected the duration of postoperative hospital stay in morbidly obese patients undergoing sleeve gastrectomy. In addition, patients with BMI ≥ 50 might be at risk of longer hospitalization after surgery.

## ﻿Findings

### ﻿Introduction

 ﻿﻿The number of patients with morbid obesity as a consequence of metabolic disease has been fewer in Japan compared with the USA or Europe, but recently, the number of such patients has increased gradually in Japan [1, 2]. Accordingly, the number of patients who have undergone bariatric surgery has also increased. These obese patients are often complicated with type II diabetes mellitus, hypertension, hyperlipidemia, ischemic heart disease, and obstructive sleep apnea. In addition, morbid obesity has serious problems in the management of anesthesia, such as difficulty in airway management, increased resistance with mechanical ventilation, and extraordinary pharmacokinetics for anesthetic or other drugs [3–5]. Furthermore, the therapeutic procedure of regional anesthesia, exemplified by epidural block, is extremely difficult in such patients. Therefore, the management of postoperative analgesia can be problematic.

In our institution, sleeve gastrectomy has been performed on morbidly obese patients since 2008, and 67 patients have undergone this procedure up to 2016. On the hypothesis that anesthetic and surgical procedures affect the duration of postoperative hospital stay, we have studied to analyze the perioperative complications and postoperative clinical course in a retrospective cohort with respect to the utility of anesthesia management, in order to prevent longer hospital stays after surgery.

## Patients and methods

This study was approved by the ethics committee of Iwate Medical University Graduate School of Medicine (no. 29-12).

Clinical data of morbidly obese patients who had undergone sleeve gastrectomy between November 2008 and December 2016 were obtained from electronic clinical records and anesthetic records.

There were no excluded patients, and 67 patients were enrolled in present study. The patients undergoing sleeve gastrectomy are discharged on postoperative day 5 on the usual condition due to a plan of the surgical department. Therefore, those subjects were divided into two groups based upon the postoperative duration of hospital stay; group S was comprised of the patients who were discharged within 5 days after surgery (*n* = 57) and group L was comprised of those who were discharged after 6 days or more (*n* = 10). We collected the data for the patients’ characteristics, anesthesia data throughout the time course, bleeding and urine volume, infused fluid volume, and perioperative complications. These data were compared between the two groups.

Statistical analyses were performed using SPSS version 22 (SPSS Inc., Chicago, IL, USA). Shapiro-Wilk test was used to assess whether the data were normally distributed. Continuous data were expressed as median (interquartile range) or mean ± standard deviations, and categorical variables were expressed as a percentage. Mann-Whitney *U* test or unpaired Student’s *t* test were used to compare continuous variables. The categorical data were assessed using a chi-square test. Logistic regression was used for the multivariate analysis. *P* values < 0.05 were considered statistically significant.

## Results

The mean duration of hospital stay was 4.8 ± 0.4 days and 7.8 ± 1.4 days in group S and group L, respectively. One patient in group L complained of chest pain on postoperative day 2. Although the diagnosis was not confirmed based upon objective findings, like a perspective view, we started heparin infusion for the prevention of ischemic heart disease and/or pulmonary thromboembolism. After that, the patient did not complain of the symptoms any further, and he was discharged at day 8 after surgery. No other severe complications occurred in any of the patients. The causes of postoperative longer hospital stay in other group L patients were as follows: a delay of poor oral intake in five cases, a lasting oozing from the intraperitoneal drain, an onset of intoxication dermatosis with the antibiotics, and a prolonged complaint of pain on the operated site in each patient.

There was no significant difference between group S and group L regarding age, gender, height, weight, and BMI (Table 1). Preoperative morbidities with complicating diseases were also similar between the groups (Table 1).

**Table 1:** Demographic profile

	Group S (*n* = 57)	Group L (*n* = 10)	*p* value
Age (years)	43.0 (19.8)	45.5 (22.8)	0.879
M/F	31/26	5/5	0.798
Height (cm)	166 ± 10.0	164 ± 10.9	0.528
Weight (kg)	121 ± 23.5	123 ± 34.1	0.893
BMI	42.0 (7.55)	43.8 (13.8)	0.681
Complications (%)			
Hypertension	82.5	80.0	0.852
Diabetes mellitus	66.7	50.0	0.311
Hyperlipidemia	77.2	50.0	0.073
IHD^a^	5.3	10.0	0.560
OSA^b^	100	80.0	0.06

Values are the median (interquartile range), mean ± SD or number (%)

^a^Ischemic heart disease

^b^Obstructive sleep apnea

All patients received remifentanil and fentanyl for pain prevention during general anesthesia. For sedation, propofol, sevoflurane, or desflurane were used at the discretion of the respective anesthesiologist. Rocuronium was routinely administered as a muscle relaxant during anesthesia, and sugammadex was used as needed.

The intra-anesthetic data are shown in Table 2. The mean durations of operation and anesthesia were slightly longer in group L than in group S, but these values were not significantly different. Although bleeding volume and infusion fluid volume were slightly larger in group L, there were no statistically significant differences in the urine output and fluid balance.

**Table 2:** Intra-anesthetic data

	Group S (*n* = 57)	Group L (*n* = 10)	*p* value
Operation time (min)	151 (55.0)	192 (180)	0.163
Anesthesia time (min)	234 (61.3)	279 (205)	0.094
Blood loss (g)	5.00 (7.50)	11.5 (35.8)	0.116
Urine output (ml)	220 (445)	201 (258)	0.502
Infusion volume (ml)	1800 (1365)	3575 (3108)	0.163
Total balance (ml/kg/h)	5.02 ± 2.29	5.79 ± 2.86	0.351

Values are expressed as the median (interquartile range) or mean ± SD

Multivariate logistic regression analysis showed anesthesia time to be predictors of a longer postoperative hospital stay (Table 3, *p* < 0.05). The reasons for our verification of regression analysis with only anesthesia time were a *p* value less than 0.1 in comparison between the groups (Table 2), and operation time, blood loss, and infusion volume were statistically significant correlated with anesthesia time (*p* < 0.05).

**Table 3:** Multivariate logistic analysis for longer hospital stay

	OR	95% CI	*p* value
Anesthesia time	0.989	0.980–0.998	0.023

## Discussion

Although laparoscopic sleeve gastrectomy in morbidly obese patients has become a routine outpatient procedure in Europe and the USA [6, 7], all our patients received perioperative treatment in the hospital for around 5 days. This difference has been considered to be due to the medical insurance systems and the consciousness of Japanese patients and their families with respect to surgical treatment. However, the most important factor is the smaller number of patients undergoing bariatric surgery in Japan. In our institution, surgically obese patients are hospitalized on the day before the operation and enter the intensive care unit soon after surgery until postoperative day (POD) 1. These patients receive walking rehabilitation after POD 1 in a general ward, and they are subsequently discharged from the hospital if they have no complications due to the operation. One patient in group L complained of chest pain on POD 2, which we suspected to be due to ischemic heart disease or pulmonary arterial emboli, and initiated heparin infusion. His complaint resolved after POD 3, and there were no abnormal findings on echocardiography and/or blood examinations. Although no other severe complications were observed in either group, a prolonged poor oral intake was observed in five patients of group L. The principal factor related to a delayed discharge was slow progress with walk rehabilitation independently due to pain at the operated site and/or muscle weakness [8–10].

**Fig. 1:**
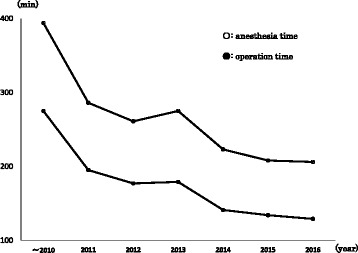
Annual changes in duration of surgery and anesthesia

In the present study, we assessed the cause of a longer hospital stay from the perspective of anesthesia management. Although the odds ratios were not particularly high, prolonged duration of anesthesia affected longer hospital stay (Table 3, *p* < 0.05). Moreover, operation time, blood loss, and infusion volume were correlated with anesthesia time (*p* < 0.05). That means the longer surgical procedure resulted in increasing of blood loss and infused fluid volume; consequently, tissue edema including gastro-intestinal tract was induced [11, 12]. Figure 1 presents the time trend of surgical and anesthetic time in our hospital. Since both durations tended to be shorter, the length of hospital stay could be stable.

There have been reports that the risk of perioperative complications is increased in patients with BMI ≥ 50 [7, 10]. In our cohort, the proportions of patients with BMI over 50 were 12% in group S and 30% in group L. That difference was not statistically significant but was slightly superior in group L (Table 4).

**Table 4:** BMI data

	Group S (*n* = 57) (%)	Group L (*n* = 10) (%)	*p* value
BMI < 40	27	30	0.808
40 ≤ BMI < 50	61	40	0.206
BMI ≥ 50	12	30	0.147

Independent chi-square test: *p* = 0.286

On the other hand, obstructive sleep apnea (OSA) has become one of the important risks for anesthetic management, especially in bariatric surgery, where the prevalence of OSA is higher and the incidences of postoperative intubation and ventilation are increased [7, 9]. In our study, the proportion of patients with the complaint of OSA was greater in group S compared with group L. Accordingly, while most of the patients with OSA have not been diagnosed preoperatively, all of our patients consulted a doctor in the department of sleep medicine prior to undergoing bariatric surgery. Therefore, postoperative complications with obstructive respiratory disorder were predicted and prevented by treatment for OSA, e.g., CPAP device.

The most important findings in this study was that longer duration of anesthesia affected the duration of postoperative hospital stay in morbidly obese patients. In addition, patients with BMI ≥ 50 may experience longer durations of hospitalization after surgery.

This retrospective study has some inherent limitations. Firstly, the number of patients was small, and the numbers in each of the groups were different. Secondly, the patients were divided into two groups based upon the duration of postoperative hospital relative to the ordinary date of discharge in our institution. However, it was unknown whether our classification is adequate or not. Thirdly, we have not analyzed the detailed anesthesia methods or postoperative analgesia.

The number of morbidly obese Japanese patients who undergo laparoscopic sleeve gastrectomy remains small. Therefore, further analyses of Japanese obese patients will be necessary in the future.

## Abbreviations

BMI: Body mass index
CPAP: Continuous positive airway pressure
OSA: Obstructive sleep apnea
POD: Postoperative day
